# Cardiometabolic Patient-Related Factors Influencing the Adherence to Lifestyle Changes and Overall Treatment: A Review of the Recent Literature

**DOI:** 10.3390/life13051153

**Published:** 2023-05-10

**Authors:** Vasiliki Kalantzi, Ioanna Panagiota Kalafati, Vasiliki Belitsi, Thomas Tsiampalis, Ioannis Koutsonasios, Odysseas Androutsos, Fotini Bonoti, Demosthenes B. Panagiotakos, Rena I. Kosti

**Affiliations:** 1 Department of Nutrition and Dietetics, School of Physical Education, Sports and Dietetics, University of Thessaly, 42132 Trikala, Greece; vkalantzi@uth.gr (V.K.); ikalafati@uth.gr (I.P.K.); vbelitsi@uth.gr (V.B.); ttsiampalis@uth.gr (T.T.); oandroutsos@uth.gr (O.A.); fbonoti@uth.gr (F.B.); 2 Department of Nutrition and Dietetics, School of Health Science and Education, Harokopio University, 17676 Athens, Greece; dbpanag@hua.gr; 3 Health Center of Pyli, 42032 Trikala, Greece; ky.pili@dypethessaly.gr; 4 Faculty of Health, University of Canberra, Canberra, ACT 2617, Australia

**Keywords:** cardiometabolic diseases, lifestyle, medication, social factors, psychological factors, cognitive factors, adherence, compliance

## Abstract

It is well acknowledged that most of the modifiable risk factors for Cardiovascular Diseases (CVDs) can be averted through lifestyle modifications beyond medication adherence. This review aims to critically evaluate the cardiometabolic (CM) patient-related factors that influence the adherence to lifestyle changes studied alone and/or in combination with medication. A comprehensive literature search of PubMed articles from 2000 to 2023 retrieved 379 articles. After removing the articles which were not relevant, a total of 28 cross-sectional studies was chosen (12 qualitative, and 16 quantitative). The findings confirmed that five groups of factors influence patients’ adherence to overall treatment: (1) health beliefs, knowledge, and perceptions regarding the risks and challenges of disease and medication intake along with adherence process perceptions; (2) self-concept; (3) emotions; (4) patient–healthcare providers relationship/communication and (5) social and cultural interactions. It is worth mentioning that cultural issues, such as culinary particularities, ethnic identity, social life as well as patients’ skills and abilities, play a profound role in the effectiveness of the recommended lifestyle modifications beyond the aforementioned common factors. The need for clear-cut culturally adapted guidelines along with personalized advice from physicians is imperative as it could improve patients’ self-efficacy. These socio-psychological factors should be seriously considered as a means to increase the effectiveness of future community prevention programs.

## 1. Introduction

Cardiometabolic diseases [CMDs, i.e., cardiovascular disease (CVD), type II diabetes mellitus, dyslipidemia, hypertension, and obesity] are one of the leading causes of mortality globally and continue to constitute a major public health problem [1].

Although the diagnosis of CMDs is not considered complicated, the effectiveness of the treatment is rather low due to patients’ compliance/adherence. When talking about “compliance” or “adherence” it should be clarified that even though these terms are used interchangeably, “compliance” has a more negative connotation, as it implies a passive behavior from the patient’s side, where the patient has to oblige to the rules. On the other hand, “adherence” is defined as the extent to which an individual’s behavior aligns with the recommendations from the health care provider, indicating a patient’s active involvement in which both doctor and patient work together [2,3,4,5].

Guidelines from the American Heart Association and the United States Department of Agriculture have very well stressed the significance of an adequate management protocol. Such a protocol will have its strong foundation on lifestyle modifications beyond medication adherence in order to manage the risk factors and control the disease [6,7].

CMDs usually require long-term adherence with the therapeutic treatment. Scientific evidence suggests that patients with CMDs struggle in their everyday life to adapt to the suggested lifestyle and drug recommendations. In the review of Ogungbe et al. on medication adherence interventions, it was outlined that a great deal of patients do not use their medication as prescribed [8]. However, results from the EUROASPIRE surveys, involving patients with coronary heart disease (CHD), indicated that patients strongly adhere to their drug regimen but do not abide to the recommended lifestyle changes [9].

Unhealthy lifestyles including nutritionally poor dietary patterns, increased alcohol consumption, smoking and physical inactivity are considered as major risk factors adding to the development and progress of CMDs [10,11]. It is well indicated that almost 90% of type II diabetes cases are preventable by maintaining a healthy body weight, eating healthy, avoiding alcohol consumption and smoking, and engaging in moderate to vigorous physical activity. As a matter of fact, most of the modifiable risk factors for CVDs could be averted through healthy lifestyle modifications [12,13,14].

According to Sabaté, there are five dimensions of adherence [15]. These include factors related to the health system, socioeconomic status, condition, therapy and patient. As regards to patient-related factors, these are associated with knowledge, beliefs, perceptions, attitudes, and expectations of the disease management and are linked with motivation, self-management, self-efficacy, self-discipline and confidence to tackle the disease [15]. All these traits increase adherence to the recommendations, profoundly benefiting patients’ health. It is therefore well-documented that overall adherence is multidimensional and complex.

Despite the extensive literature on the determinants of medication adherence [16,17,18,19,20,21], understanding and rationalizing the factors and circumstances that enhance unhealthy behaviors and non-adherence to the recommended lifestyle modifications beyond prescribed medication is challenging. In light of the above, the aim of this study was to perform, for the first time, a review that will critically evaluate studies regarding CM patient-related factors that affect the adherence to the recommended lifestyle changes, studied alone and/or in combination with medication in the same context.

## 2. Materials and Methods

### 2.1. Search Strategy

We focused on the most recent publications, only including manuscripts published after the year of 2000. For the present review, studies published in peer-reviewed journals in the English language were selected from the electronic database of PubMed via a systematic search. Studies published from 1 January 2000 up to 10 April 2023 were included. An advanced search string was developed for the selection of the articles: (“perception*” OR “belief*” OR “self-efficacy” OR “behavior*” OR “awareness” OR “emotion*” OR “feeling*” OR “knowledge” OR “experience*” OR “self-care” OR “barriers” OR “facilitators” OR “psychological” or “social” OR “financial” OR “psychosocial”) AND (“adherence” OR “compliance” OR “persistence”) AND (“cardio metabolic diseases” OR “CVD” OR “cardiovascular diseases” OR “cardiovascular risk factors”) AND (“recommendations” OR “treatment” OR “advice*” OR “change*”) AND (“lifestyle” OR “diet*” OR “physical activity” OR “habit*” OR “smoking” OR “health behavior*” OR “medication” OR “drug*”) AND (“qualitative*” OR “questionnaire*” OR “survey*” OR “interview*”).

**Figure 1:**
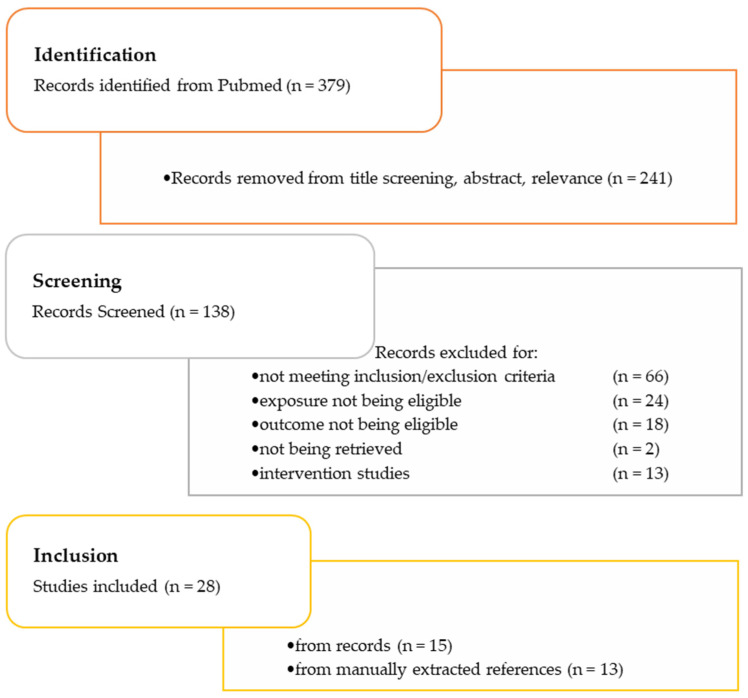
Flow chart showing the selection process of the literature search from 1 January 2000 up to 10 April 2023.

Figure 1 depicts the flow chart of the selection process of the literature search. PubMed indicated a total of 379 studies. After an initial screening of the title and the abstract, non-relevant articles were removed and finally a total of 138 studies were selected for the review in our paper. Next, we conducted a thorough scrutiny by reading the full papers and according to the inclusion criteria, 15 studies were finally retained [22,23,24,25,26,27,28,29,30,31,32,33,34,35,36]. Additionally, 13 more eligible studies were identified through manual screening, and therefore the total number of studies were 28 [37,38,39,40,41,42,43,44,45,46,47,48,49].

Papers underwent three phases of screening (titles, abstracts and full papers). Two reviewers (V.K. and V.B.) independently selected the studies, and a third researcher (R.I.K.) was consulted in order to rectify any discrepancies. Whenever the inclusion of a study was not clear, the three researchers discussed it collectively to make the final decision, keeping in mind the inclusion and exclusion criteria. All studies included were read in full by the three researchers.

### 2.2. Inclusion and Exclusion Criteria

We were primarily interested in factors affecting adherence or non-adherence to lifestyle recommendations from the patients’ perspective. Secondly, we aimed to identify patients’ viewpoints on overall adherence (lifestyle and medication). Inclusion criteria involved: (i) adults (>18 years) with CMDs or being at risk of CMDs, either self-reported or diagnosed; and (ii) a clearly defined exposure including the knowledge, awareness, beliefs, perceptions, feelings, behaviors, emotions and self-efficacy to the recommended therapeutic regimen, with an outcome related to the adherence or compliance or persistence to lifestyle changes (diet, physical activity, alcohol consumption, and smoking), and/or in combination with medication intake. Ultimately, the studies were assessed for their relevance to the aims of our review.

Studies were excluded if the exposure and/or outcome did not meet the criteria and if the outcome was only related to adherence to medical prescription. Studies including patients with mental illness were also excluded, along with institutionalized patients, patients in rehabilitation centers, patients that are currently being hospitalized, patients with an acute illness or patients who were pregnant or drug/alcohol users. Clinical trials/intervention studies, reviews, systematic reviews, and meta-analyses were also excluded from this review.

### 2.3. Data Extraction

The following relevant data were extracted from each of the selected studies: first author/year of publication, country, type of study, population characteristics (number, sex, age, and condition), objectives of the study, type of adherence to the treatment assessed, data collection tools and outcomes.

## 3. Results

### 3.1. General Characteristics of the Included Studies

A total of 28 cross-sectional studies met the inclusion and exclusion criteria and comprise the sample for this review. Table 1 shows the general characteristics of the studies included. The included studies comprise of the following designs: 12 qualitative studies [25,28,30,33,34,35,37,40,43,46,48,49] and 16 quantitative studies [22,23,24,26,27,29,31,32,36,38,39,41,42,44,45,47].

The sample size of the included studies ranged from 20 [43] to 2870 [27] participants. In all the studies, patients were over 18 years old and of both sexes, except one study which included only males [48].

The studies were conducted in the following countries between 2000 and 2022: the USA [28,44,48], South Africa [41], the UK [30,35,37,38,39,40,43], Ireland [36], Poland [47], Colombia [29], Kazakhstan [33], Iran [24], Brazil [32], Bangladesh [26], Australia [25,34], Sri Lanka [46], Nigeria [42], Zimbabwe [45], Chile [49], Netherlands [31], Kuwait [22], Taiwan [23] and Ghana [27]. The conditions mentioned in the studies are either reported specifically, such as acute coronary syndrome [29], heart failure (HF) [24,39,41], coronary artery diseases (CADs) [23], diabetes mellitus type I [36], diabetes mellitus type II [26,38,43,44,46], angina or myocardial infraction or coronary artery bypass [40], familial hypercholesterolemia (FH) [25,31], hypertension [28,42,45,47,49], and hypertension and/or hypercholesterolemia/diabetes [22,27,32,33,34,35,37,48] or as at risk of CVD [30].

Thirteen (13) studies reported data collected through individual interviews [22,25,26,27,30,34,40,41,42,43,45,46,49], while 12 studies collected data through self-reported questionnaires [23,24,28,31,35,36,38,39,42,44,45,47]. Phone interview was used in 2 studies [29,32] and 5 studies used focus groups [28,33,35,37,48]. As can be seen from above data, 4 studies used two information collection techniques [28,35,45,46].

The majority of the studies (17) assessed the adherence to overall treatment (both recommended lifestyle and medication) [23,24,25,26,27,28,29,30,39,41,42,43,44,45,47,48,49], while 11 studies assessed the adherence to at least one lifestyle change [22,31,32,33,34,35,36,37,38,40,46].

All above data are clearly outlined on Table 1.

**Table 1:** Characteristics of the included studies.

Authors	Year	Country	Type of Study	Population Characteristics (*n*, Sex, Age, and Condition)	Objectives	Data Collection Tools	Type of Adherence to Treatment Assessed	Outcome	Ref.
Thomas	2007	USA	quantitative cross-sectional study	97 adults diagnosed with HF from 2 large HF clinics (60 male, 37 female) Age (Mean ± SD): 62 ± 15	To evaluate and understand how self-concept, components of self-concept and which aspects of self-concept influence adherence to prescribed regimens (diet, exercise, and medication) in individuals with HF	Demographic Questionnaire Adherence Questionnaire Cognitive Perception of Cardiovascular Healthy Lifestyles (CPCHL) HF screening test	Diet Medication Exercise	Health regimens that are perceived by participants as threatening to self-concept are associated with lower adherence	[39]
Chiou et al.	2009	Taiwan	quantitative cross-sectional study	156 patients diagnosed with CAD (116 male, and 40 female) Age (Mean ± SD): 70 ± 10.1	To understand the modifying behaviors of CAD patients and recognize the factors that influence them	Interviews with a structured questionnaire	Lifestyle Medication	Self-efficacy was the strongest predictor of behavior influencing adherence to treatment	[23]
Ruf et al.	2010	South Africa	quantitative cross-sectional study	200 patients with chronic heart failure (CHF) from a cardiology clinic, (109 male, 91 female) Age (Mean ± SD): 56 ± 14	To investigate treatment adherence, self-care behavior and treatment knowledge in patients with CHF	Questionnaire for the examination of adherence to medications, dietary restrictions and exercise recommendations and knowledge on HF	Diet Medication Exercise	The higher the knowledge of the patients, the higher adherence to CHF management	[41]
Iyalomhe and Iyalomhe	2010	Nigeria	quantitative cross-sectional study	108 hypertensive patients for at least a year that visited health facilities, (60 male, 48 female) Age (Mean ± SD): 59.05 ± 9.06	To assess the knowledge, attitudes, perceptions and lifestyle practices of hypertensive patients as a means to ameliorate their health and adjust their treatment	In depth interview self-structured close-ended questionnaire extracting demographic characteristics and assessing knowledge, perception, and attitudes to treatment and lifestyle practices	Lifestyle (diet) Medication	The greater the impact of negative psychosocial factors on patients, the greater the non-adherence to treatment	[42]
Heydari et al.	2011	Iran	quantitative cross-sectional study	108 patients with HF, duration of at least 2 months, recruited from hospitals (67% male, and 33% female) Age: >20 years	To investigate the relationship between self-concept cognitive perception (threat and challenge) and adherence to therapeutic regimens in patients with HF	Perception of Cardiovascular Healthy Lifestyles and Adherence questionnaire	Diet and medication	Higher adherence depends on the following: higher self-consistency on the daily healthy routine patients’ view on self-concept	[24]
Singh et al.	2012	UK	qualitative cross-sectional study	20 patients with type 2 diabetes mellitus with duration approximately 10 years or more from a hospital’s diabetes care outpatient clinic (10 male, and 10 female) Median age: 60.5 years	To explore experiences of patients with diabetes as regards to their support systems and identify barriers to diabetes management (dietary and overall management)	Semi-structured face-to-face interviews that extracted demographic data and investigated barriers and the support system of patients with diabetes mellitus	Diet and Medication	Non-adherence to recommended regimens was related with the following: the feeling of dietary restrictions lack of social support fear of stigma	[43]
Walker et al.	2012	USA	quantitative cross-sectional study	378 patients with type 2 diabetes recruited from primary care clinics (117 male, and 261 female) Age: >18 years	To investigate the relation between diabetes fatalism and medication adherence and self-care behaviors (including diet, exercise and blood sugar testing) in patients with type 2 diabetes	12-item Diabetes Fatalism Scale (DFS-12) Diabetes Knowledge Questionnaire (DKQ) 4-item Morisky adherence score 11-item Summary of Diabetes Self-Care Activities (SDSCA) scale Patient Health Questionnaire (PHQ-9)	Medication Self-care behaviors (diet, exercise, blood sugar testing)	Poor adherence to medication and self-care was related with diabetes fatalism	[44]
Goverwa	2014	Zimbabwe	quantitative cross-sectional study	354 hypertensive patients on treatment from a hospital’s outpatient department with diagnosis of at least 6 months (132 male, and 222 female) Age: >18 years	To explore the prevalence of uncontrolled hypertension and the associated factors among hypertensive patients on treatment	questionnaire adapted from WHO guidelines modified 10-item Hill Bone Compliance scale 9-item scale adopted from a study conducted in Seychelles on factors affecting drug treatment compliance among hypertensives in Praslin Island Participants’ medical records	Medication Salt intake Smoking alcohol	Health education and patients’ high perception of the associated risks leads to greater adherence to the regimen	[45]
Hardcastle et al.	2015	Australia	qualitative cross-sectional study	18 patients who had received a genetic diagnosis for FH from a lipid disorders clinic involved in a genetic cascade screening program (10 male, and 8 female) Age (Mean ± SD): 50.2 ± 14.0	To investigate FH patients’ perceptions towards adherence to medication and lifestyle changes	Face-to-face interviews conducted by trained interviewer(s) with flexible design and with opportunity for follow-up questions	Lifestyle (diet, and physical activity) Medication	The lower the perceived seriousness of FH, the lower the adherence to lifestyle changes	[25]
Shawon et al.	2016	Bangladesh	quantitative cross-sectional study	144 patients with type 2 diabetes attending a tertiary hospital (101 male, and 43 female) Age (Mean ± SD): 54.4 ± 11.7	To explore the attitude towards diabetes and social and family support in patients with type II diabetes from Bangladesh	Face-to-face interview with patients using structured questionnaire	Diet Medication	The more positive the patients’ attitude towards diabetes management along with the social support, the greater the adherence	[26]
Jankowska-Polańska et al.	2016	Poland	quantitative cross-sectional study	102 patients with hypertension (50 male, and 52 female) Age (Mean ± SD): 45.5 ± 7.7	To evaluate the relationship between acceptance of illness and adherence to pharmacological and non-pharmacological therapy in hypertensive patients	Acceptance of Illness Scale (AIS) Health Behavior Inventory (HBI) Morisky Medication Adherence Scale (MMAS-8)	Pharmacological and non-pharmacological treatment	The greater the illness acceptance, the greater the adherence	[47]
Long et al.	2016	USA	qualitative cross-sectional study	34 male participants with hypertension, hyperlipidemia, or both conditions Age range: 40–65	To investigate the knowledge, attitudes and beliefs of African American men, regarding the management of hypertension and hyperlipidemia	In-person focus groups were conducted using semi-structured interview questions informed by the Health Belief Model (HBM)	Diet Medication Exercise	Self-management Barriers: medication side effects unhealthy dietary patterns Facilitators: social support positive healthcare experiences	[48]
Herrera et al.	2017	Chile	qualitative cross-sectional study	51 hypertensive patients with at least 1 month hypertensive medical treatment from two primary care public health institutions Age range: 25–80	To understand hypertensive patients adherence and non-adherence to different types of treatment programs (diet, exercise, and medication)	in-depth interviews data from patient’s medical records	Diet Medication Exercise	The higher the perceived significance of therapeutic treatment, the higher the adherence	[49]
Sarfo et al.	2018	Ghana	quantitative cross-sectional study	2870 participants with hypertension with or without diabetes from hospitals (23.2% male, and 76.8% female) Age (Mean ± SD): 58.9 ± 16.6	To investigate the causes of uncontrolled blood pressure in Ghanaian hypertensive patients focusing on improving the access to hypertension treatment	14-item version of the Hill-Bone Questionnaires on knowledge, attitudes and practices on hypertension, sources of antihypertensive medications and challenges with accessing these medications were also administered Blood pressure, weight and height were measured for each subject at enrollment	Medication Salt intake	The higher the degree to control the disease, the higher the adherence to therapeutic treatment	[27]
Espejo et al.	2019	USA	qualitative cross-sectional study	21 African American participants with essential hypertension, on single or combined oral antihypertensive regimen (24% male, and 76% female) Age (Mean ± SD): 58.1 ± 10.1	To investigate the knowledge, perception, and behaviors among African Americans with hypertension and interpret reasons behind poor blood pressure control	Structured focus group Framingham risk score men/women Treatment Self-Regulation Questionnaire (TSRQ) (exercise/diet) Charlson Comorbidity Index Behavioral Risk Factor Surveillance System Questionnaire Autonomy Preference Index High Cholesterol and High Blood Pressure Questionnaires Morisky Medication Adherence Survey National Institute of Health Daily Food List International Physical Activity Questionnaire Patient Health Questionnaire (PHQ-9) NHANES (National Health and Nutrition Examination Survey) Food questionnaire	Diet Medication	Low adherence of patients was attributed to the following: lack of trust on health care providers lack of will-power lack of knowledge on healthy behaviors stress of daily living	[28]
Henao López and Triviño Vargas	2020	Colombia	quantitative cross-sectional study	128 patients with acute coronary syndrome who underwent percutaneous coronary angioplasty in a clinic in the last 3–4 months (84 male, and 44 female) Mean age: 65.12	To investigate the relationship between adherence to secondary prevention and factors that impact the adherence in people with acute coronary syndrome, who have undergone percutaneous coronary angioplasty	Sociodemographic variables questionnaire Scale to measure therapeutic adherence for patients with chronic diseases, based on explicit behaviors Instrument to evaluate adherence by patients according to influential cardiovascular risk factors	Diet Medication	The higher the patients’ knowledge and perception of the adherence process, the higher the adherence	[29]
Alageel et al.	2020	UK	qualitative cross-sectional study	22 participants that had received a health check in the last 6 months at NHS and were assessed at medium to high risk (>10% risk) of developing CVD in next 10 years (12 male, and 10 female) Age range: 40–74	To investigate factors that possibly impact the engagement and adherence to lifestyle change interventions and medication in individuals with medium or high risk of CVD	individual semi-structured interviews	Lifestyle Medication	Low adherence was related with the following: lower understanding of CVD risk concerns over medications side-effects	[30]
Farooqi et al.	2000	UK	qualitative cross-sectional study	44 participants with high risk of heart disease that had visited general practices (24 male, and 20 female) Age: >40 years	To explore the knowledge and attitudes of individuals with high risk for heart disease as regards to the lifestyle risk factors for CHD	Focus group discussions focused on lifestyle risk factors for CHD	Lifestyle (diet and exercise)	The greater the awareness to what constitutes healthy lifestyle, the greater the adherence Lack of healthy cooking information is a barrier	[37]
Thomas et al.	2004	UK	quantitative cross-sectional study	406 diabetic patients that had visited the diabetes center of a hospital with mean diabetes duration being 10 years (224 male, and 182 female) Mean age: 56.5 years	To explore the perceived factors that prevent patients from engaging to more physical activity	Interview based questionnaires regarding: patients’ levels of physical activity underlying psychology	Physical activity	Barriers to low adherence to PA: lack of time lack of local facilities lack of willpower	[38]
Serour et al.	2007	Kuwait	quantitative cross-sectional study	334 adults with hypertension, type 2 diabetes, or both (125 male, and 209 female) Age (Mean ± SD): 53.52 ± 10.36	To understand the barriers to adherence to lifestyle changes	Structured questionnaire regarding exercise habits and barriers to compliance with diet and exercise programs	Lifestyle (diet and exercise)	Main barriers to diet adherence: socio-cultural issues Barrier to exercise adherence: lack of time comorbidities	[22]
Darr et al.	2008	UK	qualitative cross-sectional study	65 subjects from hospitals that had been diagnosed within the previous year with unstable angina or myocardial infraction, or coronary artery bypass surgery. (36 male, and 29 female) Age: >30 years	To evaluate and access the illness beliefs of patients with coronary heart disease (CHD) relating to causal attributions and lifestyle changes	Semi-structured interviews with questions about their experiences, beliefs, and understanding of their condition and the necessary lifestyle modifications	Lifestyle	The higher the patients’ health beliefs, the higher the likelihood to adopt healthier lifestyle changes	[40]
Claassen et al.	2010	Netherlands	quantitative cross-sectional study	81 participants diagnosed with FH traced in a nationwide family cascade screening program (48% male, and 52% female) Age (Mean ± SD): 48 ± 16	To investigate the perceived risk and representations of CVD and preventative behaviors of individuals diagnosed with FH via DNA testing	Self-reported data on demographic variables Illness Perception Questionnaire (IPQ-r)	Diet Physical activity	Incorrect risk perception was considered as a barrier to patients’ lifestyle changes	[31]
Ranasinghe et al.	2015	Sri Lanka	qualitative cross-sectional study	50 patients with diabetes mellitus attending medical clinics (23 male, and 27 female) Age (Mean ± SD): 61.2 ± 9.9	To examine the perception about diet and physical activity in adults with diabetes mellitus	Focus group discussions conducted by two independent observers with a focus on diet and physical activity	Diet Physical activity	The higher the wrong perception on what constitutes a healthy diet, the lower the adherence	[46]
Pizzato Galdino et al.	2016	Brazil	quantitative cross-sectional study	142 patients with cardiovascular risk who held their first nutritional consultation and had two or more cardiovascular risk factors (14.29% male, and 85.71% female) Age (Mean ± SD): 44.02 ± 13.33	To evaluate the abandonment of dietary treatments of patients with cardiovascular risk treated on an outpatient basis and explore the causes for treatment non-adherence	Telephone contact: questionnaire on the withdrawal of reasons for treatment	Diet	Non-adherence to treatment was related with the following: time restrictions patient and healthcare professional relationship	[32]
Craig and Kapysheva	2018	Kazakhstan	qualitative cross-sectional study	122 patients with CVD and Type II diabetes from various clinics and hospitals throughout two regions of Kazakhstan that were currently being treated for health conditions, such as heart disease, hypertension, diabetes (46 male, and 76 female) Mean age: 53.7	To recognize the perceived barriers to lifestyle changes	Focus group discussions accessing medical care communicating with health care providers, adherence to doctor’s recommendations.	diet and physical activity	Low adherence to therapeutic protocol was attributed to: patients’ perceptions on the dependency on doctors patients’ low level of self-efficacy patient’ ethnic identity	[33]
Gupta et al.	2018	Australia	qualitative cross-sectional study	57 participants from hospitals, clinics and community groups with self-reported type 2 diabetes and/or CVD (29 male, 28 female) Age: >18 years	To evaluate and aggregate the perceptions and experiences of participants with type 2 diabetes and/or CVD regarding disease management through diet and dietary practices	semi-structured in-depth interviews	Diet Physical activity	Adherence was linked with: greater knowledge of the disease Non-adherence was linked with: poor consultation	[34]
Moore et al.	2018	UK	qualitative cross-sectional study	67 participants (overweight or obese, with high blood pressure and/or high cholesterol) at high CVD risk from Northern Europe (27 male, and 40 female) Age (Mean ± SD): 64.00 ± 10.00	To explore the attitudes towards dietary change to the Mediterranean diet in persons with high CVD risk	Focus groups Medical information questionnaire Food Frequency Questionnaire (FFQ) based on a validated 14-item Mediterranean diet score (MDS	Diet	The higher the perceived CVD risk, the higher the adherence to dietary changes. Identified barriers: financial reasons accessibility time constraints lack of cooking skills confused nutritional media information low self-efficacy	[35]
Finn et al.	2022	Ireland	quantitative cross-sectional study	72 participants with T1DM for greater than 1 year (34 male, and 38 female) Age (Mean ± SD): 44.9 ± 12.9	To evaluate the adherence to physical activity guidelines and relating barriers, and assess the relationship between accelerometer-measured PA and CVD risk factors	accelerometer to measure physical activity self-reported International Physical Activity Questionnaire (IPAQ)	Physical activity	Low adherence to PA was attributed to the following: fear of side-effects	[36]

### 3.2. Adherence to Overall Treatment (Both Medication and Lifestyle Changes)

Several studies in the present review [23,25,27,29,30,41,42,44,45,47,48,49] indicated that higher adherence to overall treatment was related with greater knowledge and positive health beliefs, perceptions and emotions regarding disease and medication risks.

In particular, authors [27,28,29,41,47] concluded that the higher the knowledge of the patients, the higher the adherence to the recommendations for the successful management of the disease. Patients’ knowledge, education, motivation and psychological state were also highly associated with treatment attitudes and adherence [42]. More specifically, psychosocial factors, such as depression, anxiety, fear of addiction and intolerable/adverse drug effects, were associated with patients’ negative attitudes to treatment [42]. Moreover, the higher the significance that patients attribute to medication, diet and exercise, the higher the adherence to treatment regimens [49]. Similarly, in the study of Walker et al. fatalistic beliefs were shown to act as barriers to the overall effective diabetes management, [44] demonstrating that perceptions of despair, hopelessness and powerlessness contribute to low adherence to physical activity. Furthermore, illness acceptance was another crucial factor related to adherence to non-pharmacological therapy [47].

Several studies [23,24,25,26,27,28,39,43,48,49] showed that a positive self-concept as related to several other “self” constructs including self-efficacy, self-esteem, and self-consistency improve overall adherence. “Self-concept is an overarching idea we have about who we are—physically, emotionally, socially, spiritually, and in terms of any other aspects that make up who we are” [50]. Self-efficacy was concluded by Chiou et al. to be the strongest predictor of improving adherence to overall treatment recommendations [23]. Moreover, Heydari et al. indicated that higher adherence depended on higher self-consistency to the daily healthy recommended routine and on patients’ view on self-concept, including body image [24]. In other words, when self-concept is perceived as a challenge, patients tend to adhere to the therapeutic recommendations, while when it is perceived as a threat, patients do not tend to adhere. Similarly, Thomas found that health regimens that are perceived by participants as threatening to self-concept result in emotionally centered responses and non-adherent behaviors [39]. Herrera et al. also indicated that high self-esteem, autonomy, well-being and sense of freedom are correlated with higher adherence [49]. It is worth mentioning that the study of Hardcastle et al. found that patients’ low adherence to lifestyle behavioral changes was attributed to low self-control [25]. In the study of Singh et al., almost all participants felt that adherence to dietary restrictions was very strenuous [43]. In other words, as the lack of perceived control of the study participants decreased, the feelings of weakness and engagement in unhealthy behaviors enhanced [48]. Similarly, Sarfo et al. postulated that the higher the control of the disease, the higher the adherence to medication and recommended salt cessation [27].

As regards to the social aspect of the patient-related factors, findings from the present review stressed that positive social interactions, both with physicians and with the close family environment, enhance healthy long-term behavioral changes. In particular, social support, positive healthcare experiences and the core role of family have been revealed as facilitators in the adherence process [26,28,29,30,43,48]. Social and cultural pressures, such as community social etiquettes, result in families trying to conceal the disease and therefore leading to patients compromising their treatment and health as a means to avoid social stigma. Furthermore, the perceived positive impact of religion in terms of patients’ perceptions of receiving support from prayers, along with a lack of support from the healthcare providers have been identified as barriers to medication adherence [43]. Similarly, in another study [28] authors concluded that low adherence was attributed to the lack of trust on physicians’ knowledge, as well as the influence of culture and traditions on the family meals, making it difficult for some family members to follow a healthier diet [28].

### 3.3. Adherence to Recommended Lifestyle Changes (Exclusively Studied)

As regards to the patient-related factors influencing the adherence to recommended lifestyle changes, findings [22,31,32,33,34,35,37,38,40,45,46] postulated that knowledge, positive beliefs, high self-efficacy, support from friends and family along with trust on the physicians’ knowledge and ability to provide clear-cut culturally adapted guidelines and advice seem to play the most influential and beneficial role.

More specifically, the power of positive health beliefs and perceptions along with proper knowledge on what constitutes healthy lifestyle choices, the reasoning and rationalization of the necessity for the adoption of healthy lifestyle habits and their impact on the disease outcome have been revealed as the factors influencing adherence in several studies [31,34,35,36,37,38,40,46]. On the other hand, lack of information related to healthier cooking of traditional dishes and time devoted to meal preparation constitute notable barriers to leading a healthier life [22,32,37].

Self-efficacy in the adherence process has been also found to play a substantial role [22,33,38]. Specifically, low self-efficacy was a great barrier to following physical activity recommendations [33] along with limited time management skills [38]. Participants declared that even though they were well aware of the benefits and the necessity of exercising, they did not have the personal ability to make the change, while most of them preferred to take medications [33]. Another study showed that patients’ unwillingness along with their difficulty in adhering to a diet different from that of the rest of the family were found to be the barriers to the recommended lifestyle changes [22].

It is worth mentioning that socio-cultural factors [22,33,34] were revealed to be a significant determinant in the adherence process. More precisely, social gatherings and cultural issues, such as culinary particularities along with the high frequency of social events, were reported as the main barriers to diet adherence [22]. Lack of culturally appropriate advice regarding foods that hold a cultural and social importance aggravate patients’ ability to follow their dietary treatment [34]. The perception of a fixed and unchangeable ethnic identity that is linked with unhealthy behaviors, such as unhealthy dishes, has also been denoted by patients as a barrier that they cannot overcome [33].

In the present review, the fundamental role of patient–healthcare provider relationship/communication [32,33] has also been highlighted. Findings indicated that low adherence was correlated with dissatisfaction emerged from feelings that the consultation was insufficient, non-satisfactory relationship and communication with the health professional along with feelings of embarrassment in the professional’s presence [32], as well as lack of patients’ trust on physicians [33]. In other words, when patients feel that their doctor lacks knowledge, communication skills and does not properly explain the plan that needs to be followed, they feel unsatisfied and therefore they believe that their health problem cannot be tackled [33].

## 4. Discussion

This review aims to critically evaluate, for the first time, the cardiometabolic (CM) patient-related factors that influence the adherence to lifestyle changes and overall treatment. In the present work, some major influential factors of adherence and non-adherence with regards to lifestyle changes, or the combination of both lifestyle changes and drug recommendations have been highlighted. There are commonalities found in many studies on this review, regardless of the country the study took place in or the risk factor/disease studied. In particular, the findings of this review confirmed that five groups of factors influence patients’ adherence to treatment: (1) beliefs, such as knowledge, perceptions in terms of the adherence process, along with risks and challenges of disease/medication intake and health; (2) self-concept; (3) emotions; (4) patient–healthcare providers relationship/communication and (5) social and cultural interactions.

These socio-psychological factors should be seriously considered as a means to increase the effectiveness of future community prevention programs. The above mentioned factors have already been well-reported in a recently published review exclusively evaluating medication adherence from the patient’s perspective in chronic diseases [51].

Knowledge has been shown to be a common denominator for adherence both in terms of lifestyle changes and medication. Findings postulated that patients’ knowledge and accurate perception of the adherence process is of utmost importance. In order for the patient to acquire the proper knowledge, a good relationship with the healthcare provider needs to be established [52].

Communication is one of the pillars in the patient and healthcare provider relationship. Great communication creates an honest environment that is based on trust, where patients do not feel judged or embarrassed by the clinicians. It is of great importance that healthcare providers demonstrate empathy towards patients and provide health education by translating science into concise, sound lifestyle and nutrition-based guidance. Moreover, it can be relied upon the patient to make dietary choices, along with proper medicine intake whenever prescribed, which will ultimately lead to a healthy routine and a healthy life [53,54].

Patients’ trust in the knowledge of the healthcare providers is also very important, as it makes patients feel safe. The suggested guidelines will assist with the management of their disease, which will further motivate and provide confidence to the patients [55]. The psychological support that patients receive can raise their self-efficacy and help with self-management and self-discipline. This also includes support from family and friends that makes individuals feel empowered and motivated to lead a healthy life. In fact, peer influence, social stigma, lack of support from family, friends and healthcare personnel can even become stress factors and further decrease the patient’s ability or willingness to comply [56,57].

Lack of knowledge induces negative emotions with ambiguous effects, thus influencing the adherence process. This could be possibly due to fear of failure, fear of not achieving the desired outcome, or even due to concerns over medication side-effects [51,58,59]. Research has indicated that participants who postpone the thoughts of consequences, belittle the treatment and avoid unnecessary interference, have a higher likelihood to not adhere to the healthy lifestyle [60]. In particular, Urke et al. concluded that personal achievements resulting from engaging in self-management behaviors, specifically, exercise, dietary changes, medication adherence and smoking cessation, along with the close relationship with family were perceived as facilitators in the adherence process [60]. On the other hand, distrust in the medications and pharmaceutical companies as a result of the side-effects have been perceived as barriers to the medication adherence. However, despite this low medication adherence, patients expressed their willingness to increase their engagement in healthy lifestyle behaviors. Beliefs, such as the restriction of desired food groups, were perceived as a compromise in the quality of life and thus dietary restrictions were perceived as a barrier to lifestyle adherence. Wrong beliefs, such as weakness and vulnerability, are induced as a result of having a chronic disease and therefore deteriorate the adherence [60].

On the other hand, fear due to the perceived seriousness of the disease can lead to positive reinforcement towards better adherence [52].

Research has shown that diabetic patients perceive dietary restrictions as challenging to self-management, leading to negative emotions, such as frustration, depression, and anger, enhancing poor dietary self-care and thus creating a vicious cycle of poor dietary adherence and negative emotions [61]. Amankwah-Poku concluded that the apparent wavering nature of patients in regard to dietary adherence could be tackled through realization of the importance of dietary adherence, implying the crucial role of knowledge in the adherence process [61]. This finding has been consistently demonstrated in the present review. It is the patients’ illness perception, beliefs and awareness of the positive and negative impact of their medication along with their knowledge of the impact on their lifestyle behavior that determines their copying behavior and therefore their adherence and disease outcome [62]. Indeed, research has demonstrated that health regimens that are perceived by participants as threatening to self-concept result in emotionally centered responses and non-adherent behaviors [39].

Regarding lifestyle changes and more specifically dietary changes, it is worth mentioning that these are inextricably linked with cultural, religion and ethnic identity factors. This can be challenging for the healthcare provider, as it automatically means that they need to conduct an accurate screening of the patients profile in order to identify secondary or hidden beliefs and avoid providing culturally inappropriate advice. This means that they should also be well-aware and knowledgeable regarding the cultural norms in order to help patients overcome the fear of stigma deriving from social and cultural pressures, and thus create a tailored therapeutic protocol that will be feasible for the individual to adapt in their daily routine [63,64].

Moreover, results showed that there may be a bidirectional association between lifestyle and medication adherence in regard to the perceived seriousness of the disease and/or the patient’s beliefs. In particular, studies [25,60] showed that distrust of medication may increase lifestyle changes adherence, while on the other hand, the lower the perceived seriousness of the disease, the lower the adherence to lifestyle changes because patients feel that the disease could be effectively managed through medication. This observation is of utmost importance because it underlines the need for proper education delivered to both physicians and patients, highlighting that the synergistic effect of both medication and lifestyle modifications has been proven to be the most effective for disease management.

Nevertheless, it must be acknowledged that this review has several limitations. Firstly, no assessment of the included studies’ quality has been performed, which have perhaps jeopardized the validity of the findings. Moreover, there is heterogeneity between studies as regards to the size of the sample, the outcome, the age range, the assessment tools used and the location and thus no direct comparisons could be performed. It is worth mentioning that even though the vast majority of the studies were conducted in different world parts, several commonalities in influential factors have been identified. Lastly, only the PubMed database was searched, a fact which could probably limit the extent of the identified patient-related factors influencing the adherence to lifestyle changes and overall treatment of CMDs. However, many common barriers and facilitators have been stated in various studies, meaning that the possibility of missing information is limited.

## 5. Conclusions

Patients’ beliefs and perceptions, self-concept, emotions, as well as patient–healthcare provider relationship and patient social interactions appear as the catalytic components of the overall treatment adherence, acting as key factors for the successful management and tackling of CMDs. It is worth mentioning that cultural issues, such as culinary particularities, ethnic identity, and social life along with skills and abilities seem to play a profound role on lifestyle modifications beyond the other common factors. The need for clear-cut culturally adapted guidelines along with personalized advice from physicians is evident as it could improve patients’ self-efficacy.

These socio-psychological factors could provide the foundation for the design and implementation of future community prevention studies regarding lifestyle modifications in order to alleviate the burden of CMDs.

## Data Availability

Data available in a publicly accessible repository. The data presented in this study are openly available in PubMed.
